# Adaptive Expertise in Undergraduate Pharmacy Education

**DOI:** 10.3390/pharmacy11010032

**Published:** 2023-02-09

**Authors:** Naomi Steenhof

**Affiliations:** 1 Leslie Dan Faculty of Pharmacy, University of Toronto, Toronto, ON M5S 3M2, Canada; naomi.steenhof@utoronto.ca; 2 The Wilson Centre, University Health Network & University of Toronto, Toronto, ON M5G 2C4, Canada

**Keywords:** adaptive expertise, pharmacy education, cognitive integration, productive failure

## Abstract

Pharmacy educators are grappling with concerns around curriculum overload and core pharmacist competencies in a rapidly changing and increasingly complex healthcare landscape. Adaptive expertise provides a conceptual framework to guide educators as they design instructional activities that can support students on their journey towards becoming pharmacists who can perform procedural tasks efficiently, as well as creatively handle new and difficult-to-anticipate problems that arise regularly in pharmacy practice. This article explores undergraduate pharmacy education through a cognitive psychology lens and foregrounds three instructional design strategies which support the development of adaptive expertise: (1) cognitive integration, (2) productive failure, and (3) inventing with contrasting cases. These three evidence-based strategies cultivate long-term learning and provide a practical mechanism to combat curriculum overload and backwards-facing assessments. Pharmacy education can encourage the development of procedural and conceptual knowledge and position pharmacy students to excel as they move into more complicated and ambiguous roles in our healthcare system.

## 1. Introduction

Pharmacy educators locally, nationally, and internationally are examining issues of curriculum overload, core competencies for pharmacist graduates in the 21st century, and the future of pharmacy education in a world where new pharmaceutical agents are emerging daily, our understanding of the pathophysiology of diseases is expanding rapidly, and pharmacogenomics and personalized drug therapy are on our doorstep. These conversations are leading to productive debates about essential subject matter and which content should be added or removed from our curriculum. Many educators feel that we have unrealistic expectations for preserving both the breadth and depth of content within our limited curricular time, and I would agree. Additionally, I would argue that we should be spending as much time directing our attention towards which instructional strategies we are using to teach our students. Instructional design strategies that promote deep conceptual knowledge and support a strong foundation for future learning should be prioritized over coverage of broad content knowledge [1]. If students cannot form deep, integrated, conceptual knowledge about core concepts, then they may not be prepared to practice efficiently and effectively in this rapidly transforming healthcare landscape [2].

In 2020, I wrote an article entitled “Adaptive Expertise in Continuing Pharmacy Professional Development” [3]. Since that article was published, I have received many questions from pharmacy educators asking about educational strategies that support adaptive expertise development in an undergraduate education context. To summarize, the three strategies I described in the article were: explaining not just what to do but why you are doing it, allowing and encouraging struggle, and asking “what if” questions. These approaches are useful across the educational continuum; however, in that article, the application was directed to one-on-one learning in a continuing professional development context in a rural, community pharmacy. Because of the narrow focus of the article, it was difficult for educators to translate those principles into a classroom or simulation lab environment. In this article, I will describe three evidence-based instructional design strategies that pharmacy educators can use in their classrooms and simulation labs to support adaptive expertise development in undergraduate pharmacy students and trainees.

## 2. Adaptive Expertise

Adaptive expertise is an emerging model of expertise that Maria Mylopoulos and Glen Regehr introduced to the health professions education community over a decade ago [4]. This model of expertise is particularly important for educators who are training pharmacy learners who will work in environments where complexity and novelty occur regularly. Adaptive expertise may be a potential solution to these challenges because it emphasizes both the efficient management of routine, day-to-day problems (routine expertise) and also the generation of solutions to new and difficult-to-anticipate problems (adaptive expertise).

Routine expertise and adaptive expertise were initially conceptualized by two Japanese researchers, Hatano and Inagaki. They described routine expertise as a high but narrow procedural proficiency—knowing what to do, but not why one is undertaking a procedure [1]. Routine expertise is necessary for specific situations, particularly in pharmacy practice. However, a true expert must know how to identify when a problem is not routine and when that problem requires innovation (or a different way of thinking about it) to generate an optimal solution. This tenuous balance between routine and adaptive expertise means that adaptive experts not only can perform tasks efficiently, but they also can flexibly solve new problems—an essential skill for a practicing pharmacist. Too much “routine” and one risks falling into the trap of cognitive fixedness—seeing every problem as the same problem. Too much “innovation” and one will likely not complete many of the day-to-day tasks that must be finished. This is known as the “optimal adaptability corridor” (OAC) and symbolizes the balance between efficiency and innovation [5].

Although adaptive experts have many qualities, Hatano and Inagaki described three qualities in their original paper: “the ability to articulate the principles that underlie their skills, the ability to judge conventional and nonconventional versions of skills, and the ability to alter skills according to contextual changes” [1,3].

Initial research on adaptive expertise was conducted in K-12 mathematics education; however, the concept is applicable across the continuum of education and has been applied in teacher training, the military, and engineering [6]. In health professions education, the sustained interest has been on the mechanism or the processes underpinning the development of adaptive expertise (i.e., why are some students flexible with their knowledge and can translate it easily into new contexts, while others are not able to solve non-routine problems that arise in the workplace?). This body of research has led to the hypothesis that conceptual knowledge underpins expertise development and is the underlying, generalizable principle that underpins the specific context of a task, also described as “knowing why” [1,7]. Through this lens, the training in an undergraduate setting that is aimed at supporting adaptive expertise should focus on the formation of conceptual knowledge, while at the same time teaching procedural efficiency. This may sound daunting; but when instruction is aimed at the acquisition of conceptual knowledge, students have been shown to perform well on tests that assess both procedural (“knowing what”) and conceptual knowledge (“knowing why”). In short, educators can train students for both simultaneously with the same instruction and assessment strategies [5,8]. 

## 3. Aligning Instruction and Assessment to Support Adaptive Expertise

Traditional assessments tend to be ‘backwards’ facing—i.e., assessing whether students have acquired and can apply the knowledge that they have learned in our curriculum. Unfortunately, they can sometimes reward studying strategies that promote short-term performance (i.e., cramming) [9]. While students will perform well on these tests, the knowledge tends to be less connected and is usually insufficient to support long-term learning [10]. 

When we assess students for adaptive expertise and conceptual knowledge development, we may not see the benefit of our new strategies if we only test knowledge acquisition and application [11]. If we are looking to see whether students have developed a deep conceptual understanding of the material (“knowing why”) and whether they can build on that knowledge, we would be far better to place them in a knowledge-rich environment and challenge them with a new, but related, problem to see whether they can adapt their knowledge for this new context. Daniel L. Schwartz calls this an assessment of “preparation for future learning” which is “the ability to learn new information, make effective use of resources, and invent new procedures in order to support learning and problem solving in practice” [11,12,13].

These types of “preparation for future learning” tests have been used as assessment strategies for all three of the instructional design strategies that are described in this paper. Usually, these assessments involve including a new learning resource prior to a final assessment or including instructional material in the stem of the question to see how students incorporate that new knowledge into their existing knowledge [11,13]. Our goal as pharmacy educators must be to cultivate long-term, flexible learning, not short-term performance [10]. In short, our assessments should align with our teaching strategies and overall goals for our students.

## 4. Three Instructional Design Strategies to Support the Development of Adaptive Expertise

In this section, I will describe three, evidence-based instructional design approaches that have been shown through experiments to promote adaptive expertise development: cognitive integration, productive failure, and inventing with contrasting cases. I will provide a description, explain how the instructional design strategy supports expertise development, and describe a pharmacy-specific example.

### 4.1. Cognitive Integration

While pharmacy educators have valued and promoted integrative learning as a goal of their educational programs, it has not been implemented consistently across most pharmacy schools [14]. Pharmacy educators are not alone; medical educators have struggled with the same implementation challenges in their medical education curricula [15]. Much of the discourse around lack of implementation at the program level has focused on fractured curriculum, over-reliance on individual instructors, or the reality that educators are shifting the responsibility for accomplishing integration to students [15,16]. Regardless, although the development of programs that attempt to integrate basic and clinical sciences is common in health professional schools, integration at the individual teaching session is often neglected [17].

From an adaptive expertise perspective, clinical and basic science integration is beneficial when the integration occurs at the individual teaching session [18]. As Dr Woods reminds educators, the linking must occur within the mind of the learner. Cognitive integration requires that learning experiences explicitly link the “what” with the “why”, and individual teaching sessions must be carefully constructed to highlight specific connections between basic and clinical sciences [19]. In other words, clear mechanistic underpinnings must be explained and highlighted to students to optimally foster the development of expert clinical reasoning. Several studies in medicine which examine the role of biomedical knowledge in novice diagnosticians show that students who learn a causal model are better able to retain their diagnostic performance over time [20]. Another study, which used a preparation for future learning outcome measure, showed that the inclusion of basic science instruction enhanced the learning of new related content, when compared with clinically focused instruction [21]. Basic science knowledge relating causal knowledge to disease symptoms is also superior to being taught clinical knowledge along with epidemiological information after a one-week delay [22].

Cognitive integration requires that instructional materials (i.e., slides) and learning experiences (class discussions, simulation debriefs) explicitly link clinical signs and symptoms (example: of a pharmacological agent’s effects) with underlying basic science mechanisms. Again, this comes back to “knowing why”. The integration must help learners understand why a medication has a specific effect and encourage a highly connected mental model as the foundation on which new knowledge can be built [23]. Educators must deliberately choose foundational science topics that are influenced by both basic science researchers and clinicians and emphasize origins, composition, purpose, mechanisms, interactions, and consequences [24]. Recent research has shown that integration can go beyond basic science to include behavioral and sociological sciences to enhance later learning of new related concepts [25,26].

Cognitive integration of basic and clinical sciences is resource-intensive, yet important for clinical decision making in professional pharmacy practice as it promotes retention, diagnostic accuracy, and solving of complex cases [23,27,28,29]. Educators can design learning experiences based on principles of cognitive integration that allow students to efficiently master a core selection of knowledge and skills, while also preparing them for future learning that will be required by unanticipated scenarios that go beyond formal training [21].

Example:

Teaching about why opioids and benzodiazepines are toxic

Integrated example:

Opioids cause respiratory depression by activating mu-opioid receptors in the brainstem. The brainstem is where breathing control is located. This mu-opioid receptor activation causes the brainstem to be less sensitive to changes in oxygen and carbon dioxide. This reduced sensitivity means that when carbon dioxide builds up in the bloodstream, the brainstem won’t trigger respiration.

Benzodiazepines activate GABAA receptors in the brainstem which also control respiration. GABA receptors are the main inhibitory receptors in the brain. When benzodiazepines bind to GABAA, they decrease the central respiratory drive as well as brainstem responsiveness to high levels of carbon dioxide. This decrease in the respiratory drive means that respiration won’t be triggered an often.

So, the combination of opioids reducing sensitivity to CO_2_, along with benzodiazepines decreasing respiratory drive and the brainstem responsiveness to CO_2_, means that the combined inhibitory effects are especially toxic and can lead to poisoning and death.

Non-integrated example/proximate instruction.

“As doses of opioids are increased, the respiratory centre becomes less responsive to carbon dioxide, causing progressive respiratory depression. This effect is less pronounced in patients being treated for severe or chronic pain, although concurrent administration with benzodiazepines may greatly enhance this adverse effect. Respiratory depression often manifests as a decrease in respiratory rate (although minute volume and tidal exchange are also affected) and is further compounded because the cough reflex is also depressed. More recently, end-tidal capnography has become commonplace as a means to monitor opioid-induced respiratory depression, especially in those at increased risk … Caution is also urged when combining opiate analgesics with alcohol or other CNS depressants (i.e., benzodiazepines) because this combination is potentially harmful and possibly lethal” [30].

### 4.2. Productive Failure

Productive failure is a more recent instructional design strategy when compared to cognitive integration; however, it has its roots in cognitivism and desirable difficulties [9,31,32]. The originator of the term, Manu Kapur, designed a two-phase sequence where problem-solving occurs prior to instruction (PS-I) [33]. Kapur provocatively calls this design strategy productive failure, because students usually fail to arrive at the canonical answer while they are struggling to come up with a solution. Importantly, regardless of whether a student generates a correct answer or not, the students in the productive failure condition exhibit better overall learning [34]. 

From an adaptive expertise perspective, productive failure is useful in pharmacy education because it develops the conceptual knowledge students need to learn in the future. Learners are challenged to solve a problem, rather than simply being taught the correct answer. Several studies in novice pharmacy learners have shown that when compared to direct instruction or indirect failure students who learn using a productive failure methodology outperform on a “preparation for future learning” assessment, even though their performance on acquisition and application are comparable [35,36]. Lynch, Orsino, and Kawamura used a productive failure framework when studying how developmental pediatric residents developed the skill of navigating difficult conversations [37]. They found that developmental pediatric residents felt that experiencing failure was ‘productive’ in that it challenged them to “go beyond their routine approaches to create new strategies for navigating difficult conversations”. Of course, the benefit of learning through failure must be balanced with safety for the patient and family; however, residents reflected that they felt that staff physicians often over-protect residents from difficult conversations, which might limit their opportunity to receive feedback and challenge them to go beyond their existing knowledge.

These studies show that when preparing novice pharmacy students to learn new knowledge in the future, it is better to generate solutions to problems prior to instruction, rather than simply learning the correct answer, or learning about someone else’s mistakes. Researchers are still elucidating the mechanisms underlying why learning through struggle can be productive; however, the assumption is that struggling engages a depth of processing that supports the transfer of learning [10]. As Schwartz hypothesizes, giving students the “end-product of expertise too soon short-cuts the need to find the deep structure the expertise describes” [32]. When students explore unknown cases and problems, they encounter gaps in their knowledge that must be addressed to appropriately choose medication or manage an outcome.

Example

Prior to giving a lecture or leading a workshop, the instructor should pose a difficult question and ask students to try to come up with a solution. This is an example used in a 2018 study that I conducted comparing productive failure with direct instruction [35].

“You are a pharmacist who is working to be able to quickly predict creatinine clearance without collecting urine. You have collected the records of 534 consecutive patients who had two or more 24-h creatinine clearance determined at the Queen Mary Veterans’ Hospital. 96% of your patients were male. The average weight of your participants is 72 kg.

You decided to reject 29 of your patients from your study because their kidneys were not in steady state (i.e., either their kidneys weren’t producing urine because of shock, or their renal function was rapidly changing). You removed these patients because you knew that the average creatinine clearance over the 24-h period wasn’t consistent, and it would be difficult to derive a formula if the patient’s renal function was rapidly changing. Because of this you removed these patients from your analysis at this point”.

Using these variables (Table 1), invent a formula that would best approximate the creatinine clearance for your patients: Please try your best for 15 min.

It is important to give students time to ponder over the question and attempt to come up with an answer. After students fail to come up with the correct formula, the instructor must share the accepted answer (in this case, the Cockcroft Gault formula), and “why” this solution is adequate to solve the problem. At this stage, it can be very helpful to compare their partial solutions with the canonical answer to compare and contrast. Then, you can go on to give practice problems that assess knowledge acquisition, application, and preparation for future learning. This strategy not only activates students’ prior knowledge about math, physiology, anatomy, and chemistry but will prepare them to learn from the subsequent instruction.

### 4.3. Inventing with Contrasting Cases

Contrasting cases are collections of specific examples that can help learners understand the structure of a phenomenon [32]. Contrasting cases can be employed to help learners compare and contrast their exemplars or illness scripts with unique patient presentations and progressions [39]. Inventing with contrasting cases has also been described as “meaningful variation” in the literature. A helpful analogy is considering wine tasting, where the contrasts between tasting different wines side-by-side can improve knowledge and differentiation between different varieties of wine. The human brain processes information in the environment by noticing structure across variation, and perception depends on finding structure within the variability [40]. 

Similar to productive failure, education scientists have posited that when learners are told about procedures and concepts before they problem solve, it can undermine the learning of deeper structural knowledge. Schwartz and colleagues describe inventing with contrasting cases as a strategy that allows students to notice information that they might miss or overlook [32,41]. For example, in a 2011 study, participants who learned about the functional structure of ratio over a series of contrasting cases about speed and density were able to transfer that knowledge to a semantically unrelated problem several weeks later [32]. Importantly, students must go beyond developing a database of examples to support reasoning; they must understand the implications of the variation on the concept being presented [8].

One evidence-based way to do this is to ask students to explain category membership, as this has been shown to support better learning and transfer compared to thinking aloud or simply describing the instances [42]. In this study, the authors found that participants who were prompted to explain why items belonged to certain categories were more likely to induce an abstract generalization than those who were asked to describe the information [42]. For pharmacy educators, this means directing students to draw analogies across categories [43]. When students compare contrasting cases, it allows them to learn the deep structure of a problem and have a better chance of transferring the knowledge to a new, but related, problem. Notably, inventing with contrasting cases is different from productive failure, because learners tend to reinvent versions of the correct solution over 80% of the time, as opposed to productive failure where almost no learner can come up with the correct solution (which is the point) [32].

Another effective way to employ this strategy is by asking “what if” questions [3]. Although many educators use this strategy in clinical settings, some institutions (for example, Johns Hopkins) also employ meaningful variation in a structured manner when remediating struggling learners to help them build the cognitive schemas that underpin conceptual understanding and sound clinical reasoning [39]. Pusic describes that meaningful variation was helpful for residence with superficial foundation knowledge, because it is impossible to see the “breadth of all clinical presentations” during training. By using meaningful variation, faculty can support residents as they synthesize commonalities and differences in patient presentations through using divergent presentations and etiologies of common cases [39].

Example:

Propose a series of problems for a student (for example, several patients presenting with shortness of breath, coughing, and wheezing) and ask the students to think of possible explanations for the phenomena. After the students have problem-solved, then the instructor can come in with a deeper underlying structure to help students differentiate between the symptoms (SOB, coughing, wheezing) and how they could be related to the underlying causes of a COPD exacerbation compared to a CHF exacerbation. The instructor can then go on to explain why the treatment strategies are different. This is in contrast to typical teaching methods which teach CHF and COPD separately, without students having a chance to compare and contrast examples to form distinct underlying categories.

Example 2

Change the demographics of a patient to highlight key assessment and treatment plans and how they would or would not vary depending on the variable.

John is a 34-year-old man with chronic pain post-motorcycle accident who is taking oxycodone CR 20 mg orally BID and oxycodone IR 5 mg orally BID as needed. He has been taking some type of opioid since his early 20s, and finds that they are providing no relief, but causing side effects. John would like to reduce his daily dose of opioids but has been having difficulty trying to taper. He is interested in an opioid rotation to morphine and the chronic pain care team would like your recommendation on a morphine regimen.

Ask the student to present a plan with a rationale. Then follow-up with the following questions:

What if John was 84 years old? Would your plan change, and why or why not?

What if John was interested in buprenorphine-naloxone therapy? Would your plan change, and why or why not?

What if John had opioid-induced hypersensitivity? Would your plan change, and why or why not?

What if John had type 1 diabetes? Would your plan change, and why or why not?

By varying the scenarios, the instructor can highlight the importance of age on chronic pain and opioid metabolism, the efficacy and safety of buprenorphine-naloxone therapy, and how plans change if a patient is experiencing opioid-induced hypersensitivity syndrome or concomitant concerns such as a diabetes diagnosis. Variation around these concepts will allow students to develop differentiated knowledge about the concepts and understand the significance of those differences (preventing cognitive fixedness or overapplication of schemas). This also allows students to experience the breadth and depth of a clinical presentation which would be impossible to see during a clinical rotation.

## 5. Discussion

These three instructional design strategies used in pharmacy undergraduate training have the potential to promote the development of both procedural and conceptual knowledge, and better prepare our students for practice in the future. 

These strategies can be used in individual teaching sessions; however, helping learners adjust to an adaptive expertise framing requires educators who have this “shared language” and a faculty who has a goal of adaptive expertise [39]. These approaches can be challenging and more effortful for students in the moment, so it is important to help students tolerate the short-term uncertainty of not being told the correct answer immediately [32]. These strategies are in direct contrast to ‘tell-and-practice’ where the correct answer is given alongside the problem which can feel good in the moment but has the potential to promote superficial and disconnected bits of knowledge [9]. 

In addition to using these instructional strategies, educators should also focus on assessment approaches that measure not only the acquisition and application of knowledge but also “preparation for future learning” [12]. The importance of educational teaching and assessment approaches that are embedded in the cognitive and educational psychology literature is essential as pharmacy schools prepare our students for long-term learning, and for providing exceptional pharmaceutical care to our society in an ever-changing healthcare landscape.

## Figures and Tables

**Table 1:** The following table shows the age, renal function, and creatinine excretion in 249 patients [38].

Age Range Years	Mean Age Years	*n*	Mean sCr µmol/L	Mean CrCl mL/min	Mean Cr Excretion µmol/kg/24 h +/− SD
10–29	24.6	22	88	114.9	209 +/− 44.2
30–39	34.6	21	95	98.6	180 +/− 45
40–49	46.2	28	103	95.4	170 +/− 51
50–59	54.4	66	132	77.9	149 +/− 41
60–69	64.6	53	123	57.6	134 +/− 35
70–79	74.4	42	157	38.6	111 +/− 31
80–92	85.1	17	123	37.4	107 +/− 36

## Data Availability

Not applicable.

## References

[1] Hatano G., Inagaki K., Stevenson H.W., Azuma H., Hakuta K. (1986). Two courses of expertise. Child Development and Education in Japan.

[2] Carbonell K.B., Stalmeijer R.E., Könings K.D., Segers M., van Merriënboer J.J. (2014). How experts deal with novel situations: A review of adaptive expertise. Educ. Res. Rev..

[3] Steenhof N. (2020). Adaptive Expertise in Continuing Pharmacy Professional Development. Pharmacy.

[4] Mylopoulos M., Regehr G. (2009). How student models of expertise and innovation impact the development of adaptive expertise in medicine. Med. Educ..

[5] Schwartz D., Bransford J., Sears D., Mestre J. (2005). Efficiency and innovation in transfer. Transfer of Learning from a Modern Multidisciplinary Perspective.

[6] Cupido N., Ross S., Lawrence K., Bethune C., Fowler N., Hess B., van der Goes T., Schultz K. (2022). Making sense of adaptive expertise for frontline clinical educators: A scoping review of definitions and strategies. Adv. Health Sci. Educ. Theory Pract..

[7] Mylopoulos M., Kulasegaram K., Woods N.N. (2018). Developing the experts we need: Fostering adaptive expertise through education. J. Eval. Clin. Pract..

[8] Mylopoulos M., Steenhof N., Kaushal A., Woods N.N. (2018). Twelve tips for designing curricula that support the development of adaptive expertise. Med. Teach..

[9] Bjork E.L., Bjork R.A., Gernsbacher M., Pomerantz J. (2014). Making things hard on yourself, but in a good way: Creating desirable difficulties to enhance learning. Psychology and the Real World: Essays Illustrating Fundamental Contributions to Society.

[10] Soderstrom N.C., Bjork R.A. (2015). Learning Versus Performance: An Integrative Review. Perspect. Psychol. Sci..

[11] Schwartz D., Martin T. (2004). Inventing to prepare for future learning: The hidden efficiency of encouraging original student production in statistics instruction. Cogn. Instr..

[12] Mylopoulos M., Brydges R., Woods N.N., Manzone J., Schwartz D.L. (2016). Preparation for future learning: A missing competency in health professions education?. Med. Educ..

[13] Schwartz D., Bransford J.D. (1998). A time For telling. Cogn. Instr..

[14] Ratka A. (2012). Integration as a Paramount Educational Strategy in Academic Pharmacy. Am. J. Pharm. Educ..

[15] Hopkins R., Pratt D., Bowen J.L., Regehr G. (2015). Integrating Basic Science Without Integrating Basic Scientists: Reconsidering the Place of Individual Teachers in Curriculum Reform. Acad. Med..

[16] Pearson M., Hubball H. (2012). Curricular Integration in Pharmacy Education. Am. J. Pharm. Educ..

[17] Kulasegaram K.M., Martimianakis M.A., Mylopoulos M., Whitehead C.R., Woods N.N. (2013). Cognition before curriculum: Rethinking the integration of basic science and clinical learning. Acad. Med..

[18] Woods N.N. (2007). Science is fundamental: The role of biomedical knowledge in clinical reasoning. Med. Educ..

[19] Woods N.N., Mylopoulos M. (2015). How to improve the teaching of clinical reasoning: From processing to preparation. Med. Educ..

[20] Woods N.N., Brooks L.R., Norman G.R. (2007). It all make sense: Biomedical knowledge, causal connections and memory in the novice diagnostician. Adv. Health Sci. Educ..

[21] Mylopoulos N., Woods N. (2014). Preparing medical students for future learning using basic science instruction. Med. Educ..

[22] Woods N.N., Neville A.J., Levinson A.J., Howey E.H.A., Oczkowski W.J., Norman G.R. (2006). The Value of Basic Science in Clinical Diagnosis. Acad. Med..

[23] Kulasegaram K., Manzone J.C., Ku C., Skye A., Wadey V., Woods N.N. (2015). Cause and effect: Testing a mechanism and method for the cognitive integration of basic science. Acad. Med..

[24] Dahlman K.B., Weinger M.B., Lomis K.D., Nanney L., Osheroff N., Moore D.E., Estrada L., Cutrer W.B. (2018). Integrating Foundational Sciences in a Clinical Context in the Post-clerkship Curriculum. Med. Sci. Educ..

[25] Chaudhary Z.K., Mylopoulos M., Barnett R., Sockalingam S., Hawkins M., O’Brien J.D., Woods N.N. (2019). Reconsidering Basic: Integrating Social and Behavioral Sciences to Support Learning. Acad. Med..

[26] Ng S.L., Forsey J., Boyd V.A., Friesen F., Langlois S., Ladonna K., Mylopoulos M., Steenhof N. (2022). Combining adaptive expertise and (critically) reflective practice to support the development of knowledge, skill, and society. Adv. Health Sci. Educ..

[27] Baghdady M.T., Carnahan H., Lam E.W.N., Woods N.N. (2013). Integration of basic sciences and clinical sciences in oral radiology education for dental students. J. Dent. Educ..

[28] Lisk K., Agur A.M.R., Woods N.N. (2016). Exploring cognitive integration of basic science and its effect on diagnostic reasoning in novices. Perspect. Med. Educ..

[29] Cheung J.J.H., Kulasegaram K.M., Woods N.N., Moulton C.-A., Ringsted C.V., Brydges R. (2018). Knowing How and Knowing Why: Testing the effect of instruction designed for cognitive integration on procedural skills transfer. Adv. Health Sci. Educ..

[30] DiPiro J.T., Yee G.C., Posey L., Haines S.T., Nolin T.D., Ellingrod V. (2020). Pharmacotherapy: A Pathophysiologic Approach.

[31] Bjork R.A., Metcalfe J., Shimamura A.P. (1994). Memory and Metamemory Considerations in the Training of Human Beings. Metacognition: Knowing about Knowing.

[32] Schwartz D., Chase C., Oppezzo M., Chin D. (2011). Practicing versus inventing with contrasting cases: The effects of telling first on learning and transfer. J. Educ. Psychol..

[33] Kapur M., Bielaczyc K. (2012). Designing for productive failure. J. Learn. Sci..

[34] Kapur M. (2014). Productive failure in learning math. Cogn. Sci..

[35] Steenhof N., Woods N.N., Van Gerven P.W.M., Mylopoulos M. (2019). Productive failure as an instructional approach to promote future learning. Adv. Health Sci. Educ. Theory Pract..

[36] Steenhof N., Woods N.N., Mylopoulos M. (2020). Exploring why we learn from productive failure: Insights from the cognitive and learning sciences. Adv. Health Sci. Educ..

[37] Lynch J., Orsino A., Kawamura A. (2022). Productive struggle and failing safely: Implications for developing adaptive expertise in communication. Adv. Health Sci. Educ..

[38] Cockcroft D.W., Gault M.H. (1976). Prediction of creatinine clearance from serum creatinine. Nephron.

[39] Pusic M.V., Hall E., Billings H., Branzetti J., Hopson L.R., Regan L., Gisondi M.A., Cutrer W.B. (2022). Educating for adaptive expertise: Case examples along the medical education continuum. Adv. Health Sci. Educ..

[40] Paas F.G.W.C., Van Merriënboer J.J.G. (1994). Instructional Control of Cognitive Load in the Training of Complex Cognitive Tasks. Educ. Psychol. Rev..

[41] Chase C.C., Shemwell J.T., Schwartz D.L. Explaining across contrasting cases for deep understanding in science: An example using interactive simulations. Proceedings of the 9th International Conference of the Learning Sciences, ICLS ‘10.

[42] Williams J.J., Lombrozo T. (2010). The Role of Explanation in Discovery and Generalization: Evidence From Category Learning. Cogn. Sci..

[43] Gentner D., Loewenstein J., Thompson L. (2003). Learning and transfer: A general role for analogical encoding. J. Educ. Psychol..

